# Ethylene oxide exposure, inflammatory indicators, and depressive symptoms: a cross-sectional study and mediation analysis based on a non-institutionalized American population

**DOI:** 10.3389/fpubh.2024.1445257

**Published:** 2024-10-02

**Authors:** Dongru Du, Yanling Yuan, Xuan Guan, Qinglian Xie, Zaiquan Dong

**Affiliations:** ^1^West China School of Medicine, Sichuan University, Chengdu, China; ^2^Department of Pharmacy, West China Hospital, Sichuan University, Chengdu, China; ^3^Chengdu Medical College, Chengdu, China; ^4^Department of Outpatient, West China Hospital, Sichuan University, Chengdu, China; ^5^Mental Health Center, West China Hospital, Sichuan University, Chengdu, China

**Keywords:** depressive symptoms, ethylene oxide, inflammatory marker, lymphocytes, neutrophils, white blood cells

## Abstract

**Background:**

Ethylene oxide (EO) is a volatile compound positively correlated with respiratory and cardiovascular diseases. Currently, evidence suggests that environmental exposure may contribute to depressive symptoms. This study evaluated the correlation between EO exposure and depressive symptoms and investigated whether inflammatory indicators had a mediation effect on this correlation.

**Methods:**

Patients were enrolled from the National Health and Nutrition Examination Survey during 2013–2016, and 2,764 (49.67% male and 50.33% female) participants were ultimately included. EO exposure was determined by measuring hemoglobin-EO adduct (Hb-EO) concentration due to its long half-life, which was log_2_-transformed. Depressive symptoms were assessed using the Patient Health Questionnaire-9. Multivariable logistic regression analysis was performed to identify any correlations before and after covariate adjustment. Sensitivity analysis, subgroup analyses, and interaction tests were performed to further evaluate identified correlations. Mediation analysis was conducted to reveal whether specific inflammatory indicators mediated the correlation.

**Results:**

A high prevalence of depressive symptoms was observed in quartiles with increased levels of EO exposure, and male individuals exhibiting higher Hb-EO levels than female individuals. A positive correlation was observed between EO exposure and depressive symptoms (odds ratio [OR]: 1.439, 95% confidence interval [CI]: 1.310, 1.581), which remained stable even after covariate adjustment (OR: 1.332, 95% CI: 1.148, 1.545). Interaction tests showed significant effects of sex (*p* < 0.001) and thyroid diseases (*p* = 0.048) on this correlation. In the mediation analysis, white blood cell (*p* = 0.010) and neutrophil counts (*p* = 0.010) exerted a mediating effect, accounting for 13.6 and 11.9%, respectively.

**Conclusion:**

Increased exposure to EO is associated with an elevated risk of depressive symptoms, where white blood cell and neutrophil counts exert a significant mediating effect. Further prospective studies are required to investigate the potential link among EO, other environmental pollutants, and human mental health.

## 1 Introduction

Depression is a common psychiatric disease characterized by constant sadness and loss of interest (1). According to cross-sectional epidemiological studies, the prevalence of depressive disorders has reached 6.8% in China and 6.38% in Europe (2, 3). Depression is also one of the leading contributors to the Global Burden of Disease, accounting for the largest proportion (37.3%) of disability-adjusted life years among all mental disorders (4). Although the pathogenesis of depression remains under investigation, previous studies have indicated that environmental exposure may play a role in its development and progression (5–7). A retrospective study on 535 older participants revealed that the concentrations of particular phthalate metabolites were positively correlated with depression-related symptoms (5). Further, a systematic review of agricultural workers indicated a link between pesticide exposure and depression (6). Supporting these findings, a cross-sectional study on 10 volatile organic compounds showed that blood levels of benzene, 2,5-dimethylfuran, and furan were positively correlated with depression (7). Therefore, it is essential to explore the impact of environmental exposure on the pathogenesis of depression.

Ethylene oxide (EO), a reactive epoxide derived from ethylene, has been widely applied in chemical compound manufacturing and sterilization of medical equipment (8). Individuals may be exposed to EO through both endogenous and exogenous pathways. Endogenously, the human gut microbiota can convert endogenous ethylene into EO. Exogenously, additional sources of EO exposure include fruit consumption, cigarette smoking, occupational exposure, and environmental pollution (8, 9). EO can combine with DNA or proteins to form adducts, leading to cellular damage and genetic mutations (10). Furthermore, EO is known to bind to hemoglobin, forming hemoglobin-EO adducts that serve as critical biomarkers for assessing the extent of EO exposure (11, 12). Evidence suggests that chronic exposure to EO may increase the risk to a range of neurological symptoms, including memory loss, anxiety and depression (13). EO exposure is also associated with various chronic diseases such as chronic obstructive pulmonary disease (14), asthma (15), and hypertension (16). However, the relationship between exposure to EOs and depression-related symptomology remains unclear.

Inflammatory indicators, including the white blood cell (WBC) count, neutrophil count, lymphocyte count, and neutrophil-to-lymphocyte ratio (NLR), may become essential biomarkers of EO exposure. A cross-sectional study showed that various inflammatory indicators, including the total WBC, neutrophil, and lymphocyte counts, were positively correlated with EO exposure (17). Another population-based study revealed that WBC, neutrophil, and lymphocyte counts exert a mediating effect on the correlation observed between EO and asthma. As a correlation between inflammatory response and depressive symptoms has been widely recognized (18, 19), it is hypothesized that specific inflammatory indicators may play a mediating role between EO exposure and depressive symptoms.

Therefore, we conducted a cross-sectional study and a mediation analysis based on the National Health and Nutrition Examination Survey (NHANES) database. We aimed (1) to evaluate the correlation between EO exposure and depressive symptoms and (2) to investigate whether inflammatory indicators had a mediating effect on this correlation.

## 2 Methods

### 2.1 Data source and exclusion criteria

**Figure 1:**
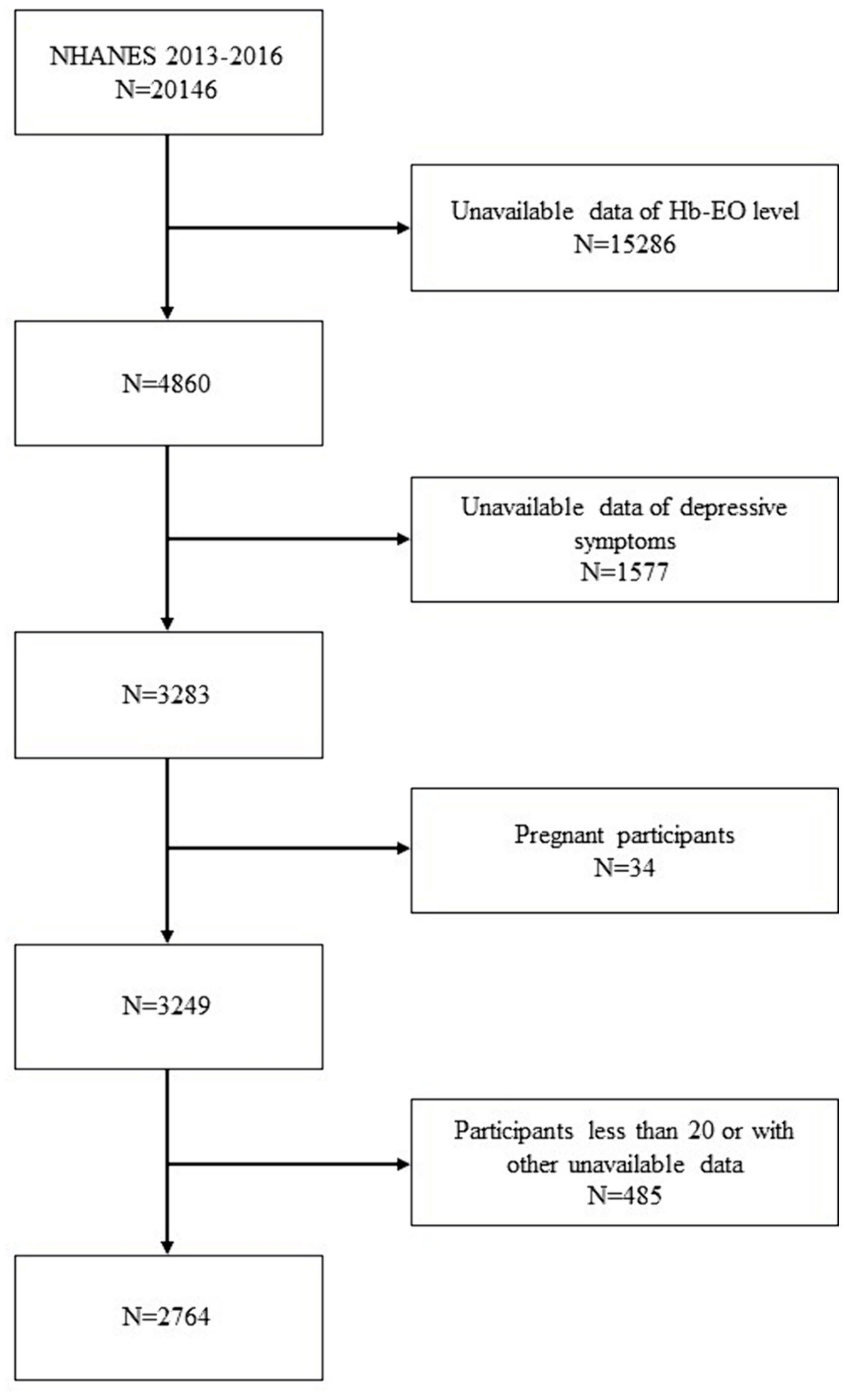
Selection process of eligible participants from the NHANES 2013–2016 database.

The NHANES is a nationally representative cross-sectional survey conducted by the National Center for Health Statistics (NCHS). A complex multistage probability sampling design was applied to select individuals who could represent a non-institutionalized American population (20). Data from the NHANES comprised demographic information, dietary data, physical examination findings, laboratory test results, and healthcare questionnaire responses. In this cross-sectional study, participants from NHANES 2013–2016 were selected based on available data regarding both EO exposure and depressive symptoms (14). The exclusion criteria were as follows: (1) unavailable EO exposure data, depressive symptom assessments, or other covariates; (2) pregnancy, and (3) participants aged <20 years. The selection process for enrolled participants is shown in Figure 1. The NHANES program was approved by the NCHS Ethics Review Board (Protocol number: Protocol #2011-17). All information concerning ethnic approval of this study can be viewed at NHANES - NCHS Research Ethics Review Board Approval (cdc.gov).

### 2.2 Measurements of EO exposure, inflammatory indicators, and depressive symptoms

All examinations were performed in the mobile examination centers during NHANES cycles. EO adducts with hemoglobin to form a hemoglobin-EO adduct (Hb-EO), which has been reported to have a longer half-life than EO *in vivo*, can serve as a sensitive indicator for EO exposure assessments (11, 12, 14). In NHANES 2013–2016, Hb-EO level was measured according to the modified Edman reaction, which forms Edman products under alkaline or neutral conditions with the effect of N-alkylated amino acids. The Hb-EO assessment process consisted of four parts: (1) Specimen preparation: At the NHANES mobile examination center, blood was collected, processed, and aliquoted into vials, then stored at −30°C in preparation for Hb-EO level measurements; (2) Assessment of total hemoglobin in the sample solution: The total Hb levels were determined via photometric measurements accompanied by rigorous quality assessments; (3) Modified Edman reaction and Edman product isolation: This reaction was used to identify the Hb-EO adducts and prepare them for analysis; and (4) Edman product analysis and result processing via high-performance liquid chromatography and tandem mass spectrometry. All measurements were conducted based on a well-established procedure in clinical chemistry [ETHOX_H (cdc.gov); the standardized methods can be found at 2016_MEC_Laboratory_Procedures_Manual.pdf (cdc.gov)].

The blood samples for inflammatory indicators, including total WBC, neutrophils, lymphocytes, and NLR, were also collected in the mobile examination center and analyzed via a complete blood count with 5-Part differential and counted using the UniCel DxH 800 Analyzer [Complete Blood Count (cdc.gov)] (21). NLR was calculated by dividing the neutrophil count by the lymphocyte count.

Depressive symptoms were evaluated using the Patient Health Questionnaire-9 (PHQ-9), a 9-item questionnaire that determines the frequency of depressive symptoms experienced during the most recent 2 weeks (11, 22). The response for each question included “not at all,” “several days,” “more than half the days,” and “nearly every day,” which was scored 0, 1, 2, and 3, respectively. A total score of ≥10 was defined as the presence of depressive symptoms.

### 2.3 Covariates

Covariates were selected based on prior research and available NHANES data (11, 14). Demographic factors, including sex, age, race, education status, marital status, and economic status (represented by the poverty-to-income ratio [PIR]), are essential characteristics to include. Moreover, given the demonstrated impact of body composition on mental health, body mass index (BMI) was included. Comorbidities such as hypertension, diabetes (including HbA1c levels), cancers, and thyroid diseases were also considered. Lifestyle factors, including measured nicotine levels to assess smoke exposure and annual alcohol consumption (more/less than 12 drinks/year), were included to evaluate drinking status.

### 2.4 Ethics approval

This population-based study included human participants, which was approved by the Ethics Review Board of NCHS [NHANES - NCHS Research Ethics Review Board Approval (cdc.gov)]. Written informed consent were obtained from all individuals in this survey.

### 2.5 Statistical analyses

All statistical analyses were conducted according to the primary sample units and strata of the NHANES. An appropriate subsample weight was also applied according to the NHANES recommendation [ETHOX_H (cdc.gov)]. Continuous variables are presented as mean with standard estimate (SE) and were analyzed using the *t*-test, and categorical variables are presented as percentages with SE and were analyzed using the chi-square test.

Due to the skewed distribution of Hb-EO levels, log_2_Hb-EO was applied to reach a normal distribution. Multivariate logistic regression analysis was performed using the three models to explore the correlation between EO exposure and depressive symptoms. In Model 1, no covariates were adjusted for. Model 2 was adjusted for sex, age, and race only. Model 3 was adjusted for all covariates mentioned in Section 2.3. Sensitivity analysis was conducted by dividing log_2_Hb-EO into four quartiles to test the stability and trend of this correlation. Subgroup analyses with interaction tests was also performed for all categorical variables to further investigate correlations. Multivariate logistic regression was applied again to reveal the role of inflammatory indicators in the relationship between EO exposure and depressive symptoms. Mediation analysis, including multiple regression analysis, path analysis, mediation model construction, and the bootstrap method, was performed to explore whether inflammatory indicators were involved in EO’s effect on depressive symptoms (23). The results of mediation analysis were presented as total effect, direct effect, mediation effect, and mediation proportion. All statistical analyses were performed using the R and Empower software (EmpowerStats | Data Analysis for Biostatistics & Epidemiology), and a *p*-value of < 0.05 was considered statistically significant.

## 3 Results

### 3.1 Summary of the key findings

Participants with higher levels of EO exposure exhibited a higher prevalence of depressive symptoms. Multivariate logistic regression suggested that EO exposure increased the risk of depressive symptoms, even after adjusting for covariates. Interaction tests revealed that sex and a history of thyroid diseases had remarkable interacting effects on this association. Additionally, mediation analysis showed that both WBC and neutrophil counts mediated the association between EO exposure and depressive symptoms.

### 3.2 Baseline characteristics of the enrolled participants

A total of 2,764 (49.67% male and 50.33% female) participants were selected for enrollment from NHANES 2013–2016. Table 1 shows the baseline characteristics of the eligible participants in the four log_2_Hb-EO quartiles; there were significant differences among the quartiles, except for hypertension (*p* = 0.9360) and cancer prevalence (*p* = 0.5253). The log_2_Hb-EO ranges for quartiles 1, 2, 3, and 4 were 2.54–3.91, 3.91–4.39, 4.40–5.41, and 5.42–10.35, respectively. The prevalence rates of depressive symptoms in quartiles 1, 2, 3, and 4 were 4.08, 4.54, 5.39, and 15.95%, respectively (*p* < 0.001). Moreover, male individuals exhibited higher levels of log_2_Hb-EO than female individuals (*p* = 0.003) (Supplementary Table S1).

**Table 1:** Weighted baseline characteristics of eligible participants enrolled in this study (divided by different quartiles of EO exposure).

	Total	Q1 (2.54–3.91)	Q2 (3.91–4.39)	Q3 (4.40–5.41)	Q4 (5.42–10.35)	*p*-value
Sex (%)						0.0022
Male	49.67 (1.35)	46.01 (2.63)	45.83 (2.00)	52.56 (2.62)	56.25 (2.27)	
Female	50.33 (1.35)	53.99 (2.63)	54.17 (2.00)	47.44 (2.62)	43.75 (2.27)	
Age (%)						<0.0001
<60	71.77 (1.38)	72.85 (1.86)	64.93 (1.98)	67.65 (2.49)	82.15 (1.96)	
≥60	28.23 (1.38)	27.15 (1.86)	35.07 (1.98)	32.35 (2.49)	17.85 (1.96)	
Race (%)						<0.0001
Hispanic	13.81 (1.92)	12.45 (2.21)	15.03 (2.34)	17.63 (2.89)	10.65 (1.54)	
Non-Hispanic White	67.90 (2.77)	75.81 (3.16)	69.51 (3.23)	56.00 (4.60)	66.69 (2.60)	
Other races	18.28 (1.76)	11.75 (1.74)	15.46 (2.02)	26.37 (2.86)	22.66 (2.16)	
Education (%)						<0.0001
Under high school	13.33 (1.24)	8.63 (1.45)	10.34 (1.49)	13.51 (2.01)	22.82 (2.16)	
High school or equivalent	21.08 (1.00)	18.15 (2.26)	16.52 (1.45)	22.02 (2.26)	29.37 (2.83)	
Above high school	65.59 (1.71)	73.22 (2.42)	73.14 (2.00)	64.48 (3.10)	47.81 (2.71)	
Marital status (%)						0.0060
With partner	62.82 (1.36)	64.02 (2.43)	68.77 (2.80)	62.50 (2.85)	54.60 (2.84)	
Widowed, divorced, or separated	18.15 (1.03)	17.78 (1.92)	13.58 (1.67)	18.11 (1.82)	24.03 (2.30)	
Never married	19.03 (1.25)	18.21 (2.22)	17.65 (2.47)	19.38 (1.95)	21.37 (2.00)	
PIR (%)						<0.0001
PIR < 1.3	21.51 (1.51)	13.37 (1.73)	15.79 (1.69)	24.80 (2.62)	35.78 (3.22)	
1.3 ≤ PIR < 3.5	36.29 (1.49)	36.28 (2.61)	36.48 (2.56)	32.06 (2.54)	39.97 (3.08)	
PIR ≥ 3.5	42.20 (2.35)	50.34 (3.60)	47.73 (3.32)	43.13 (3.17)	24.25 (2.60)	
BMI (%)						0.0183
BMI < 25	26.91 (1.49)	25.40 (3.05)	22.34 (2.34)	28.32 (2.38)	32.94 (2.51)	
25 ≤ BMI < 30	33.57 (0.94)	30.29 (2.38)	37.35 (2.99)	35.46 (2.09)	31.70 (2.06)	
BMI ≥ 30	39.51 (1.36)	44.31 (2.67)	40.31 (2.91)	36.22 (2.26)	35.36 (2.49)	
Hypertension (%)						0.7941
Yes	32.21 (1.49)	33.55 (2.14)	30.50 (2.15)	32.49 (2.48)	32.21 (3.13)	
No	67.79 (1.49)	66.45 (2.14)	69.50 (2.15)	67.51 (2.48)	67.79 (3.13)	
Diabetes (%)						0.0066
Yes	14.14 (1.07)	11.08 (1.71)	16.45 (2.17)	18.11 (1.64)	11.78 (1.56)	
No	85.86 (1.07)	88.92 (1.71)	83.55 (2.17)	81.89 (1.64)	88.22 (1.56)	
Depressive symptoms (%)						<0.0001
Yes	7.17 (0.62)	4.08 (0.68)	4.54 (0.86)	5.39 (0.98)	15.95 (1.74)	
No	92.83 (0.62)	95.92 (0.68)	95.46 (0.86)	94.61 (0.98)	84.05 (1.74)	
Cancer (%)						0.5202
Yes	11.25 (0.96)	12.75 (1.74)	11.27 (1.66)	11.01 (1.25)	9.49 (1.84)	
No	88.75 (0.96)	87.25 (1.74)	88.73 (1.66)	88.99 (1.25)	90.51 (1.84)	
Thyroid disease (%)						0.0381
Yes	12.76 (0.95)	15.86 (1.93)	13.13 (1.56)	10.43 (1.44)	10.43 (1.63)	
No	87.24 (0.95)	84.14 (1.93)	86.87 (1.56)	89.57 (1.44)	89.57 (1.63)	
Alcohol consumption (%)						<0.0001
Yes	78.12 (1.32)	80.51 (1.73)	72.04 (2.89)	74.41 (2.32)	85.56 (1.17)	
No	21.88 (1.32)	19.49 (1.73)	27.96 (2.89)	25.59 (2.32)	14.44 (1.17)	
Serum cotinine (ng/mL)	62.85 ± 4.27	22.87 ± 6.40	17.69 ± 6.48	27.27 ± 6.68	200.85 ± 8.05	<0.0001
Glycohemoglobin (%)	5.62 ± 0.03	5.49 ± 0.05	5.63 ± 0.05	5.79 ± 0.05	5.6 ± 0.005	0.0004

Categorical variables are presented as the percentage (standard error [SE]). Continuous variables are presented as mean ± SE.

Q, quartile; BMI, body mass index; PIR, poverty-to-income ratio.

### 3.3 Association between EO exposure and depressive symptoms

Table 2 shows the results of multivariate logistic regression analysis of the correlation between EO exposure and depressive symptoms. Positive correlations were observed among the crude (odds ratio [OR]: 1.439, 95% confidence interval [CI]: 1.310, 1.581), part-adjusted (OR: 1.455, 95% CI: 1.321, 1.603) and fully adjusted models (OR: 1.332, 95% CI: 1.148, 1.545). Compared with quartile 1 (although quartiles 2 and 3 in the three models did not reach significance), quartile 4 showed a significantly higher risk of depressive symptoms in the three models. The *p*-values for the trends in the three models were all <0.05.

**Table 2:** Association between ethylene oxide exposure and depressive symptoms.

Hb-EO	Crude model	Part-adjusted	Fully adjusted
Continuous	1.439 (1.310, 1.581) <0.001	1.455 (1.321, 1.603) <0.001	1.332 (1.148, 1.545) 0.001
Q1	Ref	Ref	Ref
Q2	1.119 (0.626, 1.999) 0.707	1.121 (0.629, 1.997) 0.702	1.220 (0.633, 2.354) 0.494
Q3	1.339 (0.770, 2.328) 0.311	1.394 (0.783, 2.481) 0.271	1.291 (0.676, 2.466) 0.378
Q4	4.464 (2.909, 6.850) <0.001	4.756 (3.078, 7.350) <0.001	3.230 (1.789, 5.833) 0.002
*p* for trend	<0.001	<0.001	0.002

Hb-EO, hemoglobin-ethylene oxide adduct.

Crude model: No covariates was adjusted.

Part-adjusted: Adjusted for sex, age, and race.

Fully adjusted: Adjusted for sex, age, race, education, marital status, poverty-to-income ratio, body mass index, hypertension, diabetes, cancers, thyroid diseases, alcohol consumption, and nicotine and HbA1c levels.

### 3.4 Subgroup analyses and interaction tests

**Figure 2:**
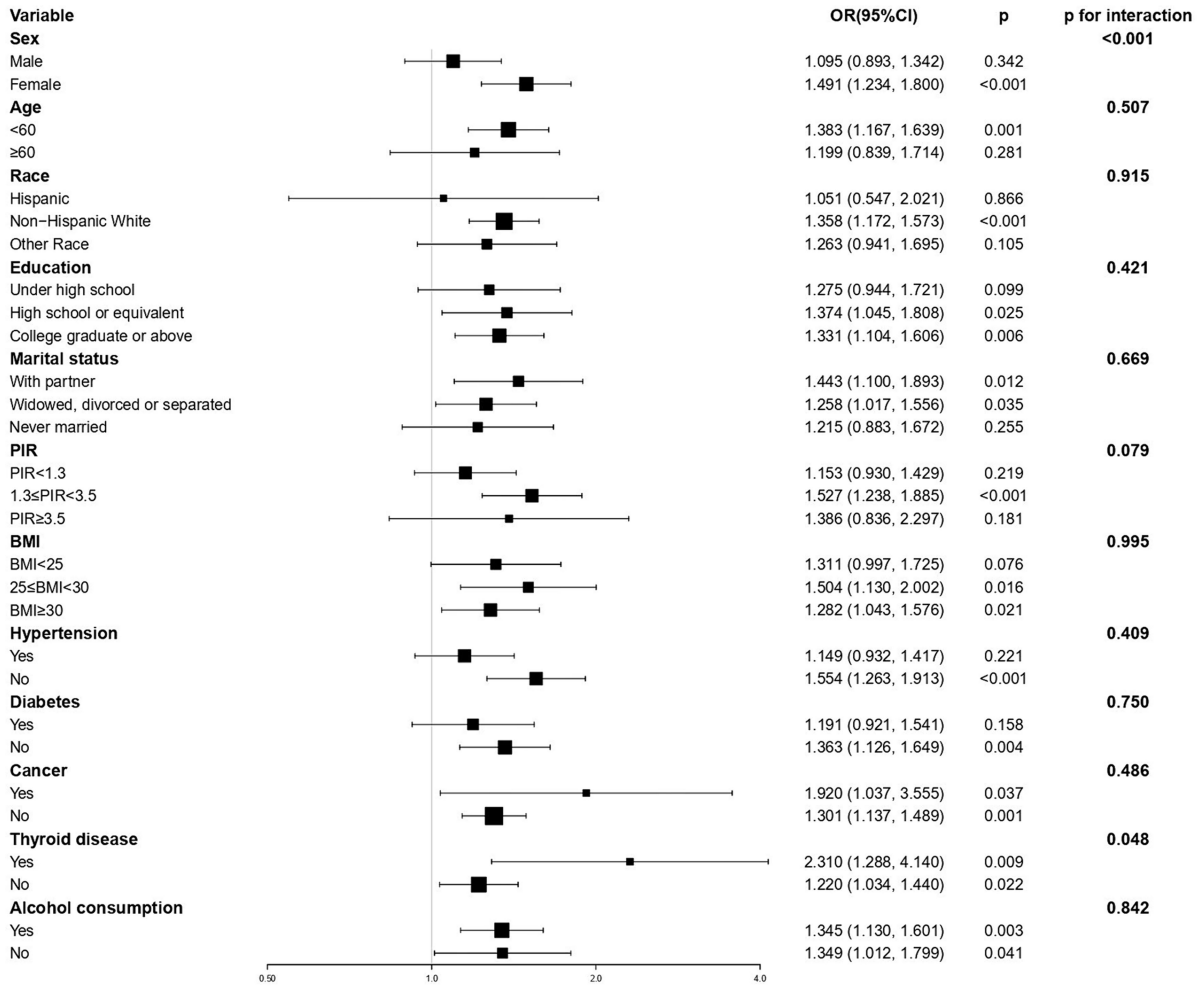
Subgroup analyses of the association between ethylene oxide exposure and depressive symptoms. All covariates (sex, age, race, education, marital status, PIR, BMI, hypertension, diabetes, cancer thyroid disease, alcohol consumption, and level of nicotine and HbA1c) were adjusted in subgroup analyses. PIR, poverty-to-income ratio; BMI, body mass index.

The results of the subgroup analyses are shown in Figure 2. The significant correlation between EO exposure and depressive symptoms was not significant in certain subgroups, including male sex, age ≥ 60 years, race (except non-Hispanic White), education status (under high school education), never married, PIR (<1.3 or ≥3.5), BMI <25 kg/m^2^, or history of hypertension or diabetes. The interaction tests showed that sex (*p* < 0.001) and thyroid diseases (*p* = 0.048) had a significant impact on the correlation between EO and depressive symptoms.

### 3.5 Mediation analysis of inflammatory indicators

**Figure 3:**
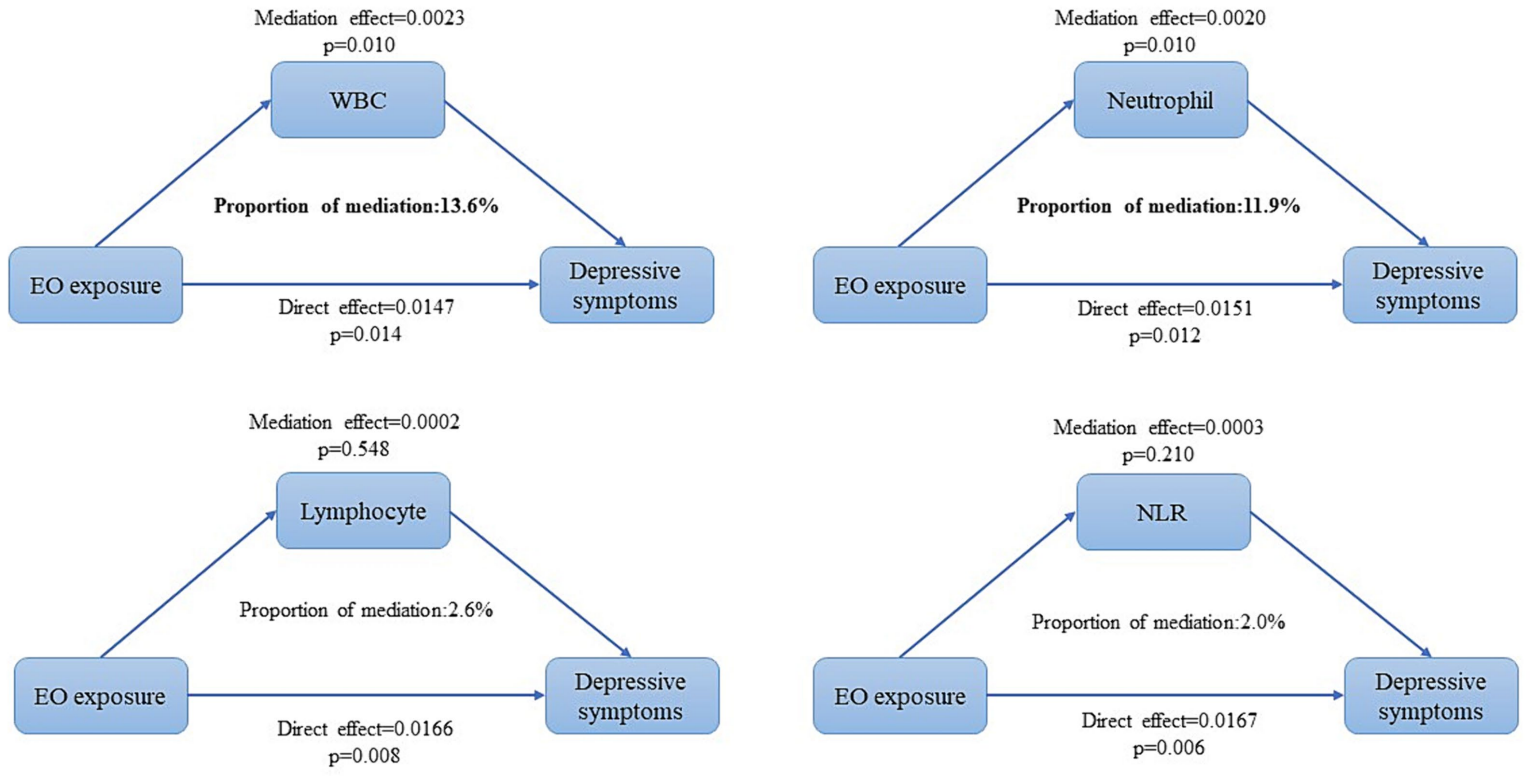
Mediation analysis of WBC, neutrophil, and lymphocyte counts and NLR over the association between EO exposure and depressive symptoms. EO, ethylene oxide; WBC, white blood cell; NLR, neutrophil-to-lymphocyte ratio. All covariates (sex, age, race, education, marital status, poverty-to-income ratio, body mass index, hypertension, diabetes, cancer thyroid disease, alcohol consumption, and levels of nicotine and HbA1c) were adjusted in mediation analyses.

Table 3 shows the correlations between Hb-EO levels and different inflammatory indicators using multivariate logistic regression analysis. Hb-EO level was positively correlated with WBC (*β* = 0.374, 95% CI: 0.266, 0.482), neutrophil (β = 0.257, 95% CI: 0.175, 0.339), and lymphocyte (β = 0.089, 95% CI: 0.055, 0.123) counts. However, no significant association was observed between Hb-EO and NLR (β = 0.043, 95% CI: −0.005, 0.091) after covariate adjustment. Mediation analysis was then applied to explore whether inflammatory indicators played a mediating role in the association between EO exposure and depressive symptoms. As shown in Figure 3, WBC (*p* = 0.010) and neutrophil (*p* = 0.010) counts significantly mediated this correlation, accounting for 13.6 and 11.9%, respectively. However, the mediating effects of lymphocyte count (*p* = 0.548) and NLR (*p* = 0.210) were not statistically significant.

**Table 3:** Association between ethylene oxide exposure and inflammatory indicators after covariate adjustment.

Variables	β (95% CI)	*p*-value
WBC	0.374 (0.266, 0.482)	<0.001
Neutrophil	0.257 (0.175, 0.339)	<0.001
Lymphocyte	0.089 (0.055, 0.123)	<0.001
NLR	0.043 (−0.005, 0.091)	0.070

CI, confidence interval; NLR, neutrophil-to-lymphocyte ratio; WBC, white blood cell.

Adjusted for sex, age, race, education, marital status, poverty-to-income ratio, body mass index, hypertension, diabetes, cancers, thyroid diseases, alcohol consumption, and nicotine and HbA1c levels.

## 4 Discussion

This cross-sectional, population-based study provides evidence that EO exposure is positively correlated with depressive symptoms. Moreover, it is identified that some inflammatory indicators, including WBC and neutrophil counts, exerted mediating effects on this correlation.

### 4.1 Potential sources of EO exposure

Humans are exposed to EO through a variety of unavoidable endogenous and exogenous sources within the general population (8). Previous evidence has suggested that ethylene may act as a potential precursor to EO (8). The consumption of fruit may increase EO exposure via metabolizing dietary ethylene into ethylene oxide (24, 25). A preliminary study suggested that fruit store workers exhibited a 23 pmol/g increase in Hb-EO levels (26). Moreover, some ethylene-producing bacteria have been detected in the human gastrointestinal tract, suggesting that the gut microbiota may also result in EO formation (8). Smoking can also increase the chance of EO exposure due to the incomplete combustion of cigarette smoke (8). Other evidence suggests that EO exposure may result from occupational sources because EO is applicable in medical equipment sterilization (8). As EO may be produced after the burning of vegetation or degeneration of agricultural waste, environmental pollution may become another source of EO exposure (9). However, although the aforementioned sources have been identified as potential contributors to EO exposure, further quantitative details are necessary to determine the amount of exposure from each potential source.

As a biomarker for quantifying EO exposure, Hb-EO has shown promising reliability. Walker et al. reported that after 4 weeks of EO exposure, Hb-EO formation was linear at concentrations ranging from 3 to 33 ppm and increased in slope at concentrations above 33 ppm in mice (27). Another human-based study suggested that Hb-EO levels were significantly elevated in smokers, aligning quantitatively with the exposure source (28). These findings highlight the reliability of Hb-EO in reflecting EO exposure accurately.

### 4.2 Hypothesis of how EO exposure affects the pathogenesis of depression

**Figure 4:**
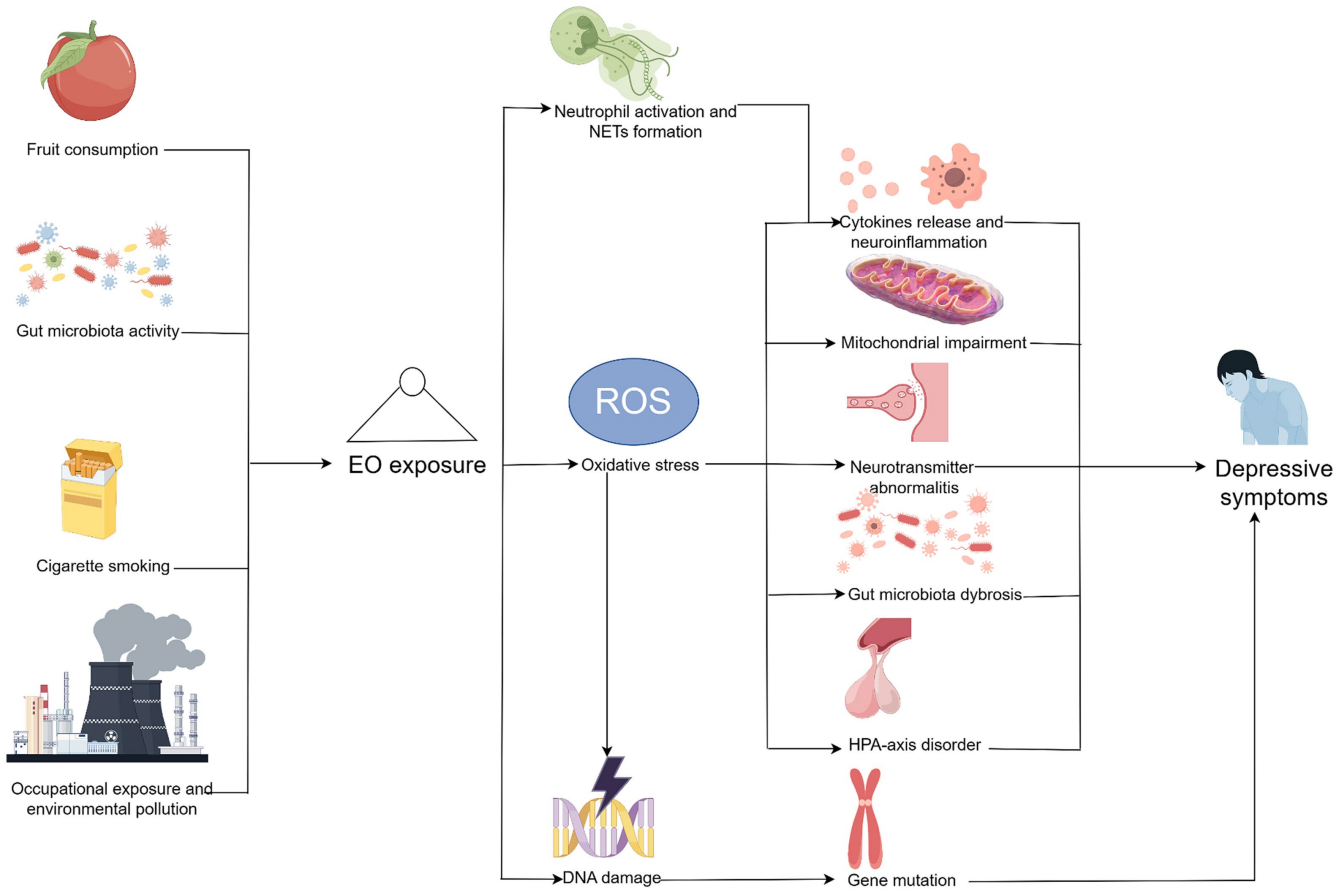
Potential mechanisms underlying the impact of ethylene oxide on depressive symptoms. Created by Figdraw. Further studies are needed to verify these pathways.

The possible mechanisms underlying the impact of EO on depressive symptoms are summarized in Figure 4. Mediation analysis showed that WBC count accounted for 13.6% of the mediation proportion, whereas the neutrophil count accounted for 11.9%, indicating that neutrophils may play an essential role on this correlation. Previous evidence suggested that neutrophils accelerate the formation of neutrophil extracellular traps in response to various extrinsic or intrinsic stimuli (29). Another study revealed that the formation of neutrophil extracellular traps aggravates pro-inflammatory cytokine release and promotes neuroinflammation in mice, which subsequently cause further depressive symptoms (30). Therefore, exposure to EO may promote neutrophil activation and neutrophil extracellular trap formation, subsequently leading to neuroinflammation and depressive symptoms. However, this hypothesis warrants further investigations as other potential inflammation-related factors, such as infection, metabolic diseases, and immunological diseases, may also affect the pathogenesis of depression through inflammatory responses. Moreover, these inflammatory indicators may only reflect a certain aspect of inflammation.

Moreover, oxidative stress may serve as an essential contributor to depressive symptoms as a consequence of EO exposure. Compared with controlled rats, rats exposed to EO showed elevated levels of malondialdehyde and advanced oxidation protein products and lower levels of superoxide dismutase, glutathione, and catalase, suggesting the existence of oxidative stress under EO exposure (9). Oxidative stress may contribute to depressive symptoms through multiple pathways, including neuroinflammation, mitochondrial impairment, neurotransmitter abnormalities, gut microbiota dysbiosis, and hypothalamic–pituitary–adrenal disorders (31). Oxidative stress promoted pro-inflammatory cytokine (such as interleukin-6 and tumor necrosis factor-*α*) release, leading to dysregulation of the inflammatory response and cell damage (32). Consistent with this, Liu et al. reported decreased levels of malondialdehyde, which is regarded as a biomarker of oxidative stress, in stress-exposed mice after muscone (an active ingredients from the musk) treatment (33). Meanwhile, the exposed mice showed decreased inflammatory levels and more adaptive behavioral responses compared with those that were not treated (33). Oxidative stress was also associated with overproduction of reactive oxygen species (ROS) in mitochondria, causing enzyme inactivation and mitochondrial dysfunction, which may promote depression-related symptomology (34, 35). Salvi et al. also suggested that air pollutants can induce oxidative stress and mitochondrial impairments, thereby disrupting neural circuitry and leading to depressive-like behaviors (36). Moreover, oxidative stress is closely associated with alterations in neurotransmitter function. For example, accumulation of excessive ROS may inhibit glutamate transporter 1 expression in astrocytes and affect glutamine synthetase levels, leading to glutamate excitotoxicity and subsequent depressive symptoms (31). Oxidative stress may also produce neurotoxic substances via the oxidation of tryptophan, which is a precursor of serotonin and is associated with depression pathogenesis (32). Furthermore, dysbiosis of the gut microbiota may be a consequence of excessive oxidative stress. Current evidence suggests that gut-brain axis disturbance may serve as a vital downstream pathway of oxidative stress, affecting the abundance and function of the microbiota (37). Since the gut microbiota is also responsible for regulating oxidative stress, its dysfunction may further promote oxidative stress, contributing to the pathogenesis of depression (38). Furthermore, oxidative stress may disturb activity of the hypothalamic–pituitary–adrenal axis. A clinical trial in adolescent children with depression suggested that morning cortisol concentration was positively correlated with lipoperoxide expression, while the aldosterone concentration was positively correlated with 8-isoprostane (*p* = 0.019), suggesting that stress hormone secretion is positively correlated with oxidative stress in adolescents with depression (39). Another study reported that corticosterone administration promoted deficits in the forced swim test in rodents and contributed to the development of depressive-like behaviors, such as anhedonia and learned helplessness (40).

Additionally, the increased incidence of genetic mutations caused by exposure to EOs may promote depressive symptoms. EO-DNA adducts, including N3-hydroxyehyladenine and O6-hydroxyethylguanine, are regarded as the initial steps in the development of genetic mutations (8, 10). N3-hydroxyethyladenine may adduct with the minor groove of the DNA helix, whereas O6-hydroxyethylguanine directly affects the base pairing of nucleotides (10). Evidence suggests that genetic mutations may also contribute to depressive symptoms. A study on patients with Alzheimer’s disease suggested that a carboxypeptidase E/neurotrophic factor-α1 gene mutation in humans leads to impaired memory acquisition and depressive-like symptoms in transgenic mice (41). Another study on patients with primary aldosteronism uncovered that *KCNJ5* mutations were associated with higher baseline PHQ scores and a better response to treatment with adrenalectomy, indicating that KCNJ5 mutations may participate in the formation of depressive symptoms (42). Moreover, subsequent effects of EO exposure, including oxidative stress, promote genetic mutations. A comparative study showed that patients with major depressive disorder had greater mitochondrial oxidative damage (*p* < 0.001) and lower leukocyte mtDNA copy numbers (*p* = 0.037) (43). Therefore, genetic mutations may also contribute to depressive symptoms resulting from EO exposure.

### 4.3 Interpretation of interaction test results

The results of this cross-sectional study also showed that sex (*p* < 0.001) and history of thyroid diseases (*p* = 0.048) significantly affected the association between EO exposure and depressive symptoms. We also observed that the significant association disappeared in male participants after subgroup analysis. This may be attributed to more EO exposure and less depressive symptoms in male individuals than in female individuals. Current evidence suggests that higher concentrations of Hb-EO adducts are detected in males than in females, which may be explained by a higher smoking prevalence in male individuals (8). By contrast, the prevalence of depressive disorders in female individuals is significantly higher than that in male individuals (44). Regarding the interactive effect of thyroid diseases, a history of thyroid diseases may make patients more vulnerable to EO exposure and more susceptible to depressive symptoms (45). However, the specific role of thyroid diseases in the association between EO exposure and depressive symptoms warrants further investigations.

### 4.4 Strengths and limitations of this study

This study has some strengths that should be discussed. By investigating the correlation between EO exposure and depressive symptoms and the mediating role of inflammatory indicators, this study suggested that reduction of EO exposure may become a feasible way of preventing depressive disorders. Moreover, as EO exposure has been linked to an increased risk of chronic respiratory diseases (14), cardiovascular diseases (16), and metabolic diseases (46), this study may also draw additional attention to reveal impact of environmental factors on various psychiatry diseases. Additionally, due to the public health implications of EO exposure, this study may prompt ethical considerations that could influence public health policies. However, there are still some limitations that are worth mentioning. First, owing to the cross-sectional design, we could not determine a causal relationship between EO exposure and depressive symptoms (47). Second, although we conducted covariate adjustments in the part-adjusted and fully adjusted models, potential confounders (such as those unavailable in the NHANES database) may still affect the study results. Third, the diagnosis of depressive symptoms was based on self-reported PHQ-9 scores, which could introduce bias. Fourth, since the NHANES program focused on the non-institutionalized American population, the generalizability of the aforementioned findings warrants further investigations. Fifth, further details of EO exposure (such as specific amounts and durations that lead to increased levels of Hb-EO) remain limited and warrant further investigations. Sixth, although both laboratory tests and the PHQ-9 questionnaires were completed at the mobile examination centers, the precise intervals between these tests for each individual were not available, which may also lead to potential bias.

## 5 Conclusion

This cross-sectional study demonstrated that EO exposure (measured by Hb-EO adduct) positively correlated with depressive symptoms. Inflammatory indicators, including WBC and neutrophil counts, exerted a mediating effect on this correlation, while the mediating effects of lymphocytes and the NLR remained insignificant. Further research should investigate the underlying mechanisms linking environmental pollutants to mental disorders. Such studies may offer new insights into the prevention of mental diseases.

## Data Availability

The raw data supporting the conclusions of this article will be made available by the authors, without undue reservation.
