# Supplementary material for: Ethylene oxide exposure, inflammatory indicators, and depressive symptoms: a cross-sectional study and mediation analysis based on a non-institutionalized American population
Source: Front Public Health. 2024 Oct 2;12:1445257. doi: 10.3389/fpubh.2024.1445257 (PMC11480028; doi:10.3389/fpubh.2024.1445257)
Supplement: Supplementary file 1 [file Table_1.DOCX]

Table S1 Comparison of Hb-EO level between male and female

|  | Male | Female | p |
| --- | --- | --- | --- |
| Hb-EO (pmol/g Hb) | 63.51 (4.07) | 53.96 (4.31) | 0.146 |
| Log_2_Hb-EO | 4.88 (0.06) | 4.65 (0.06) | 0.003 |

Categorial variables are presented as the percentage (standard error)
